# Evaluation of the neuroprotective and antioxidant effects of *Dorema aucheri* extract on cerebral ischaemia-reperfusion injury in rats

**DOI:** 10.1080/13880209.2019.1597132

**Published:** 2019-04-07

**Authors:** Javad Rasouli Vani, Mohammad Taghi Mohammadi, Mahsa Sarami Foroshani, Elham Rezazade

**Affiliations:** a Neuroscience Research Center Baqiyatallah University of Medical Sciences, Tehran, Iran;; b Department of Physiology and Biophysics School of Medicine, Baqiyatallah University of Medical Sciences, Tehran, Iran

**Keywords:** Brain infarction, brain swelling, oxidative stress, nitrosative stress

## Abstract

**Context:** The hydroalcoholic extract of *Dorema aucheri* Bilhar (Umbelliferae) (DA) leaves, a medicinal plant, has powerful antioxidant properties.

**Objective:** This study evaluates the neuroprotective effects of pre-treatment with DA leaves extract against cerebral ischaemia-induced brain injury through alteration of the antioxidant capacity.

**Materials and methods:** The study was conducted in three groups of Wistar rats (N = 47) as follows; sham, control ischaemic and pre-treated ischaemic groups. Rats were administered a fresh hydroalcoholic extract of DA leaves at a dosage of 200 mg/kg/day for 14 days. Then, the middle cerebral artery (MCA) of the right hemisphere was occluded for 90 min to achieve cerebral ischaemia. After 24 h reperfusion, cerebral infarction and superoxide dismutase (SOD) and catalase activities, as well as malondialdehyde (MDA), glutathione, and NOx contents were determined in the right hemispheres.

**Results:** Occlusion of the right MCA caused noticeable cerebral infarction (298 ± 21 mm^3^) in control ischaemic group, but pre-treatment with DA extract considerably attenuated it (92 ± 14 mm^3^) in the pre-treated ischaemic group. DA extract significantly decreased the levels of MDA by 28% and NOx by 11% in pre-treated ischaemic group compared to the control ischaemic group. DA extract also enhanced glutathione content by 7%, SOD activity by 16% and catalase activity by 46% in pre-treated ischaemic rats compared to control ischaemic rats.

**Discussion and conclusions:** DA is able to improve the antioxidant capacity and injuries of ischaemic brain. It is proposed as a neuroprotectant following cerebral ischaemia to decrease the injuries of ischaemic stroke.

## Introduction

Ischaemic stroke is the second or third leading cause of death worldwide (Rodrigo et al. 2013). Cerebral ischaemia activates several neurodegenerative pathways such as inflammation, apoptosis, free radicals generation and oxidative stress (Shi and Liu 2007; Rodrigo et al. 2013). Generation of various free radicals during the ischaemic stroke is the primary mechanism of damage and neurodegeneration. It is involved in all stages of the ischaemic injury, from the intravascular events after the cessation of cerebral blood flow and occurrence of ischaemia to the reperfusion phase (Chen et al. 2011; Rodrigo et al. 2013). Reactive oxygen species (ROS) are highly active molecules that combine with cellular biomolecules such as proteins, DNA, and lipids, pairing with their single electrons and resulting in oxidation of these molecules (Gill and Tuteja 2010). Based on gene expression studies, an increase in generation of ROS in the ischaemic brain can activate gene expression of several neurodegenerative factors such as apoptotic signals and pro-inflammatory cytokines that exacerbate the ischaemic injury (Chen et al. 2011; Rodrigo et al. 2013). ROS can also combine with nitrogen free radicals causing peroxynitrite production, as a potent oxidative radical that results in protein nitration and dysfunction (Chen et al. 2011). On the other hand, these free radicals are removed by the non-enzymatic antioxidant (glutathione) and antioxidant enzymes (such as superoxide dismutase and catalase) in a healthy brain (Slemmer et al. 2008; Gill and Tuteja 2010). These antioxidant defence systems are weakened after the occurrence of brain ischaemia when oxygen free radicals are overproduced by activation of the pro-oxidant enzymes (Kinouchi et al. 1991). Several pro-oxidant enzymes such as NADPH-oxidase and xanthine oxidase, which contribute to the ROS generation, are activated in ischaemic stroke (Chan 2001; Miller et al. 2006; Ishizuka et al. 2008). Therefore, potentiation of the enzymatic and non-enzymatic antioxidant systems of the ischaemic brain would be the potential strategy for stroke therapy.

*Dorema aucheri* Bilhar (Umbelliferae) grows in cold mountainous regions in southern provinces of Iran at the beginning of the spring season. *Dorema aucheri* is reported to be a medicinal plant (Khanahmadi et al. 2012), and is consumed as herbal medicine, but there are only a few reliable studies about the advantages or possible side-effects of *D. aucheri* extract. It is regularly used largely by the people of central areas of Iran to control diabetes mellitus, to decrease the blood triglycerides and to modulate pain. The lipid-lowering and antidiabetic effects of the hydroalcoholic extract of *D. aucheri* leaves have been reported in nicotinamide-streptozotocin induced type 2 diabetic rats (Ahangarpour et al. 2014). Khanahmadi et al. (2012) also demonstrated other properties of the *D. aucheri* extract including antioxidant and antimicrobial effects. Based on available literature, *D. aucheri* extract contains a significant amount of antioxidant agents such as flavonoids, anthocyanins and phenolic acid (Mianabadi et al. 2015). The presence of these compounds, particularly flavonoids, is associated with free radical scavenging and antioxidative property of *D. aucheri* extract (Roghani et al. 2004). Additionally, other beneficial effects of *D. aucheri* extract have been reported on thyroid hormones (Azarneushan et al. 2010), antioxidant enzymes (Khoshvaghti et al. 2013), the haematologic system (Mokhtari et al. 2008) and also serum levels of testosterone, FSH and LH (Ghasemiboroon et al. 2014), in several pathophysiological states.

Since free radicals generation and weakening of the brain’s antioxidant defence system play a major role in the pathophysiology of ischaemic stroke, we examined the possible neuroprotective and antioxidant effects of pre-treatment with hydroalcoholic extract of *D. aucheri* leaves against ischaemia-induced brain damage in the rat model of ischaemic stroke. Additionally, we analyzed the possible protective mechanisms of this extract through potentiation of the enzymatic and non-enzymatic antioxidant defence systems during cerebral ischaemia-reperfusion injury.

## Materials and methods

### Animals

The protocols of the present study were done based on the acknowledged standards of animal care and use approved by the Institutional Care and Use of Animals Committee of Baqiyatallah University of Medical Sciences. Forty-seven male Wistar rats, weighing ∼280–320 g, were purchased from the animal house facility center of the University of Baqiyatallah Medical Sciences. The rats were kept in a standard situation (one rat in a cage) with a controlled light period (12 h light/dark cycle), relative humidity (60%) and temperature (23 ± 1 °C), and also *ad libitum* access to the rat chow and water.

### Preparation of hydroalcoholic extract of *D. aucheri* leaves

*Dorema aucheri* leaves were collected around the Zagros mountain ranges at the beginning of spring season (April 2017) by the local people according to a voucher specimen. After drying the leaves in shade on the ground, an electrical grinder was used to powder the *D. aucheri* leaves. Then, the maceration method was applied to prepare the extraction. Macerating the powder was performed by using 70% ethanol and 30% water for 72 h at room temperature. Whatman filter paper (No. 1) was used to filter the mixture. After centrifuging the filter (3000 rpm, 20 min), the supernatant was evaporated at ambient temperature. Then, the extract was dried at room temperature and kept at 4 °C until used. The obtained semisolid mass was freshly used for the daily treatment of rats.

### Development of brain ischaemia-reperfusion

**Figure 1.**
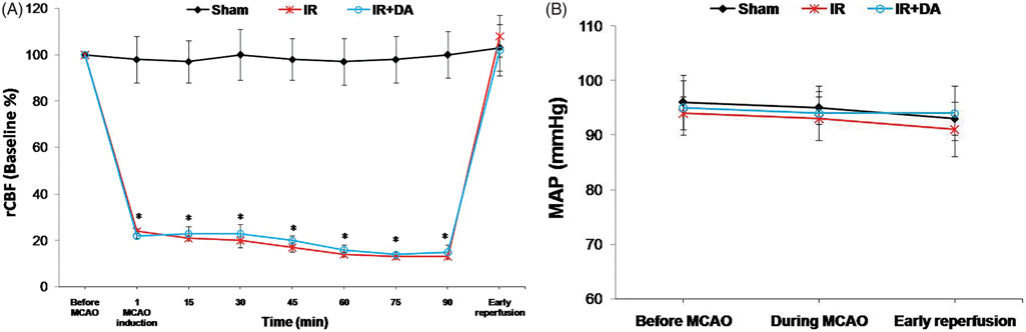
Representative changes of the regional cerebral blood flow (rCBF) at ischaemic zones and mean arterial pressure (MAP) before MCAO, during MCAO and early reperfusion periods. All data are presented as mean ± SEM. All values are in physiological range except to rCBF during MCA occlusion in control ischaemic (IR) and treated ischaemic (IR + DA) groups. *As significant difference compared to sham group at same time (*p* < 0.05).

In the present study, we used the intraluminal filament method to achieve brain ischaemia-reperfusion by the middle cerebral artery occlusion (MCAO) in the right hemispheres of the ischaemic rats, which previously described in detail by Longa et al. (1989). First, the rats were anesthetized with isoflurane (4% induction, 2.5% maintenance), (Forane, UK). Surgery was performed in the dorsal recumbent position. The core temperature of animals was continuously recorded and maintained at 37 ± 1 °C by using a heating pad and lamp during the surgery. After exposing the right common carotid artery and also the right external and internal carotid arteries through a midline incision in the neck area, a 4 cm poly l-lysine-coated nylon thread (3–0) was inserted into the internal carotid artery via the external carotid artery. The prepared filament smoothly advanced until a feeling of resistance was met and seeing a sharp decline in the blood flow trace, which was recorded using a laser Doppler flowmeter (AD Instrument, Model: ML191, Australia). There was a 75–85% reduction in regional cerebral blood flow (rCBF) of ischaemic zones during the MCAO (Figure 1). After 90 min MCAO, the reperfusion phase was initiated by gently taking out of the filament to re-establish the blood flow to the ischaemic areas (Vani et al. 2016). All incisions were ultimately sutured and the rats were recovered from anaesthesia.

### Experimental protocols and grouping

The surgery was performed in the rats of sham group (*n* = 11) at the neck area without induction of the middle cerebral artery (MCA) occlusion. The rats of control ischaemic group (IR, *n* = 12) underwent same surgery at the neck region as the sham group to achieve MCAO. Then, brain ischaemia-reperfusion was induced by 90 min occlusion of the MCA followed by 24 h reperfusion. The rats of the pre-treated ischaemic group (IR + DA, *n* = 11) received the *D. aucheri* extract orally for 14 days (200 mg/kg/day in distilled water), and then brain ischaemia-reperfusion was done. Other procedures were followed in the same way as the control ischaemic rats. After termination of the surgical procedures, the rats returned to a warm cage for recuperation during the reperfusion period. The rats that survived during the 24 h reperfusion period are the number of animals presented for each group (Table 1).

**Table 1.** The number of rats in each group that was distinctly used for assessment of each parameter.

Groups	Cerebral infarction and swelling		Biochemical assessments		Total rats	Died rats	Mortality (%)
	Total	Died	Total	Died			
Sham	6	0	5	0	11	0	0
IR	12	5	9	4	21	9	43
IR + DA	8	2	7	2	15	4	26

### Evaluation of brain infarct volume

Based on the method of 2, 3, 5-triphenyl tetrazolium chloride (TTC, Sigma) staining, we evaluated the brain infarct volume.

In brief, the brains were separated under deep anaesthesia, cleaned, and solidified by immersing in pre-cooled normal saline (4 °C). Then, six slices were prepared from each brain using a brain matrix. The slices were stained with TTC solution (2%) and fixed in 10% buffered formalin solution. After staining, the colour of the non-ischaemic areas was red and the ischaemic area was white. The slice images were digitized by using a Canon camera. Images of the stained sections were taken. Grossly visible infarction zones were quantified using image analysis software (NIH Image Analyzer). Cerebral infarct volume for each hemisphere was calculated by the sum of infarct sizes for six slices and multiplying by 2 (thickness of each slice) (Darabi and Mohammadi 2017).

The following formula was used to calculate the corrected infarct volume for oedema. Finally, the values of brain infarction were expressed as mm^3^.

Correctedinfarctvolume= Left hemisphere volume– (Right hemisphere volume – Measured infarct volume)

### Determination of tissue swelling for ischaemic hemispheres

The percent of tissue swelling for ischaemic (right) hemisphere was performed as followed; first, the total volume of each hemisphere (right and left) was determined by the sum of hemisphere sizes for six slices and multiplied by 2 (thickness of each slice). Then, the percent of tissue swelling was calculated using the following formula:

Tissue swelling (%)= [(VRight Hemisphere− VLeft Hemisphere)/VLeft Hemisphere]×100

### Evaluation of brain antioxidant capacity and oxidative damage

Ischaemic (right) hemispheres were quickly removed under deep anaesthesia for evaluation of the antioxidant parameters and oxidative damages. First, the ischaemic hemispheres were weighed and washed in an ice-cold phosphate buffer saline (PBS). After homogenization of the hemispheres in ice-cold PBS (1:10), the homogenized solutions were centrifuged at 14000 *g* at 4 °C for 15 min. Then, the supernatants were used to determine the brain contents of glutathione (GSH), malondialdehyde (MDA) and nitrate as well as the activities of superoxide dismutase (SOD) and catalase.

The method of Bradford (1976) was used to quantify the protein content of each hemisphere for calculation of data.

### Glutathione levels of the ischaemic hemispheres

According to the method of Tietz (1969), the glutathione levels of ischaemic hemispheres were assessed. First, by adding sulfosalicylic acid (5%), the cellular protein was precipitated. After centrifugation of the solution at 2000 *g* for 10 min, the supernatant was removed and glutathione level was assayed as follows:

Hundred microlitres of the protein-free supernatant of the cell lysate, 100 µL of 0.04% 5,5′-dithiobis-(2-nitrobenzoic acid) (DTNB) in 0.1% sodium citrate and 800 µL of 0.3 mM Na_2_HPO_4_. After 5 min, the DTNB absorbance was recorded at 412 nm. Based on the measurement sensitivity, the standard curve for glutathione was done between 1 and 100 µM (Mohammadi et al. 2013).

The glutathione levels of ischaemic hemispheres were calculated as nmol/mg protein.

### SOD activity of the ischaemic hemispheres

According to the capability of SOD to inhibit the reduction of nitroblue tetrazolium (NBT), the enzyme activity was measured using the method of Winterbourn et al. (1975).

First, potassium phosphate buffer (0.067 M and pH 7.8) was added to 0.1 M EDTA containing 0.3 mM sodium cyanide, 1.5 mM NBT and 0.1 mL of sample. Then to initiate the reaction, riboflavin (0.12 mM) was added to each sample. After 12 min incubation, the absorbance of samples was recorded by a spectrophotometer (UV 7500, Spectro Lab, England) at an excitation of 610 nm for 5 min. The amount of enzyme needed to induce 50% inhibition was taken as 1 U (Mohammadi et al. 2013).

The activity of the SOD enzyme in the ischaemic hemispheres was calculated as U/mg protein.

### Catalase activity of the ischaemic hemispheres

The method of Aebi (1984) was used to determine the activity of catalase in tissue homogenate. First, the homogenate was incubated in the reaction mixture that contained 0.1 mL homogenate and 0.85 mL potassium phosphate buffer (50 mM and pH 7.0) at room temperature for 10 min. Then, the reaction was begun by adding 0.05 mL H_2_O_2_ (30 mM prepared in potassium phosphate buffer 50 mM and pH 7.0). A decrease in the absorbance was recorded by a spectrophotometer at an excitation of 240 nm for 3 min. The specific activity of catalase was calculated as 1 µmol H_2_O_2_ decomposed U/mg protein.

### MDA assay in the ischaemic hemispheres

In the current study, the method of Satoh (1978) was utilized for MDA assay. First, 1.5 mL of trichloroacetic acid (TCA, 10%), was added to 0.5 mL of tissue homogenate. The mixture was vortexed and incubated at room temperature for 10 min. Then, 2 mL thiobarbituric acid (0.67%) were added to 1.5 mL supernatant and incubated in a boiling water bath for 30 min in sealed tubes. After cooling the samples to room temperature, 1.25 mL *n*-butanol was added and vortexed. The samples were finally centrifuged at 2000 *g* for 5 min and the supernatants were separated. The absorbance of the solutions was recorded by a spectrophotometer at an excitation of 532 nm. 1, 1, 3, 3-Tetraethoxypropane was used as a standard to determine the levels of MDA (Abbassi et al. 2016). The MDA levels of the ischaemic hemispheres were calculated as nmol/mg protein.

### NOx (nitrate and nitrite contents) assay in the ischaemic hemispheres

To determine the nitrosative damage of ischaemic hemispheres, the colorimetric reaction method (Griess reagent) was used. First, the homogenate (0.1 mL) was deproteinized by adding zinc sulphate (0.2 mL). The mixture was centrifuged at 4000 *g* and 4 °C for 20 min to separate supernatant. Then, 0.1 mL vanadium III chloride was added to the 0.1 mL of supernatant (as a sample) or pure water (as blank) or sodium nitrite (as standard) to reduce nitrate to nitrite. After adding the 0.05 mL sulphanilamide (0.01%) and 0.05 mL *N*-[1-naphthyl] ethylenediamine dihydrochloride (NED, 0.01%), the mixture was incubated at 37 °C for 30 min in dark place. Ultimately, the absorbance of the solutions was recorded by a spectrophotometer at the excitation of 540 nm. To determine a standard curve for calculation of the nitrate concentration, the sodium nitrate solution was used in the different concentrations (Mohammadi 2016). The nitrate levels of the ischaemic hemispheres were expressed as nmol/mg protein.

### Statistical analysis

All data in the current study were expressed as mean ± SEM. Data were analyzed with analysis of variance (ANOVA) followed by Tukey *post hoc* test. All states, differences were considered significant if *p* < 0.05.

### Histopathological assessment

Twenty-four hours after surgery in sham group and 24 h after termination of ischaemia in ischaemic groups, animals were transcardially perfused with normal saline and followed by 4% PBS-buffered formaldehyde. The brains were removed and post-fixed in 4% PBS-buffered formaldehyde for 48 h. After fixation and tissue processing, coronal serial sections (5 μm in thickness) were prepared for conventional histologic examination. Paraffin-embedded sectioning (each 50 μm intervals), processed routinely for Cresyl violet (CV) staining. The histological changes were observed through a light microscope (Nikon, Japan). Eosinophilic neurons were defined as intensely acidophilic, triangular shape, major darkening and shrinkage of nucleus and cytoplasm.

## Results

### The pre-treatment effects of *D. aucheri* extract on cerebral infarction

**Figure 2.**
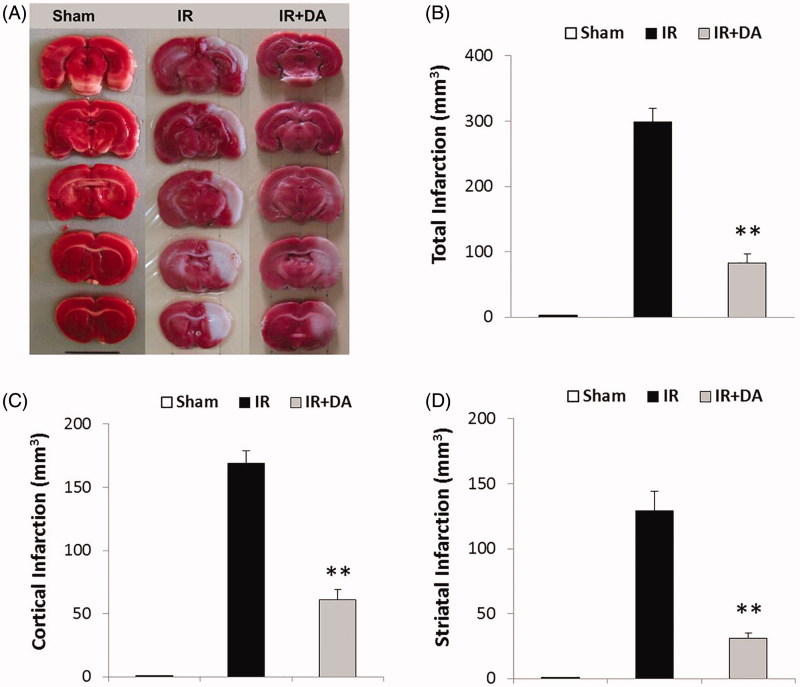
Photograph (A) shows the coronal sections of the brains stained with 2, 3, 5-triphenyl tetrazolium chloride (TTC) at the end of the experiment in sham rats, control ischaemic group (IR) and ischaemic rats treated with *Dorema aucheri* extract (IR + DA). Ischaemic zones are coloured white, whereas non-ischaemic zones are stained red (black). The graphs show the quantified total (B), cortical (C) and striatal (D) infarct volume (mm^3^) for mentioned groups at the termination of the experiment. All values are expressed as mean ± SEM. **As significant difference compared to IR group (*p* < 0.001).

Figure 2(A) shows the photographs of TTC-stained coronal sections from sham, control ischaemic and *D. aucheri*-treated ischaemic group. No infarction was observed in right or left hemispheres of sham rats. Occlusion of MCA developed extensive lesions (white zones) in large areas of both cortex and striatum in the right hemispheres of control ischaemic rats. As shown in Figure 2(A), the cerebral infarction considerably decreased in *D. aucheri*-treated ischaemic group compared to control ischaemic group.

Occlusion of MCA in the right hemispheres of control ischaemic rats developed cerebral infarction both in cortex and striatum by 169 ± 10 mm^3^ and 129 ± 15 mm^3^, respectively (Figure 2(C,D)). The mean value of total infarct volume in control ischaemic group was 298 ± 21 mm^3^ (Figure 2(B)). *Dorema aucheri*-pre-treated ischaemic rats showed a significant decrease in the cerebral infarction both in the cortex (61 ± 8 mm^3^) and striatum (31 ± 4 mm^3^) compared to control ischaemic group (*p* < 0.001). Also, the mean value of total infarct volume in *D. aucheri*-pre-treated ischaemic rats was 92 ± 14 mm^3^ (Figure 2(B)).

### The pre-treatment effects of *D. aucheri* extract on tissue swelling

**Figure 3.**
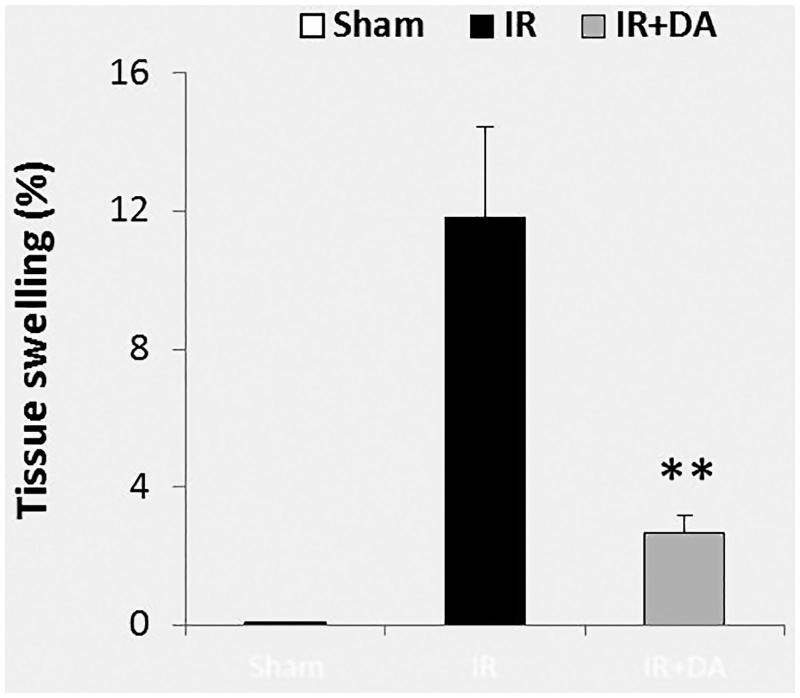
The graph shows the percent of tissue swelling in the ischaemic (right) hemispheres of sham rats, control ischaemic group (IR) and ischaemic rats treated with *Dorema aucheri* extract (IR + DA) at the end of the experiment. All values are expressed as mean ± SEM. **As significant difference compared to IR group (*p* < 0.01).

The calculated percentage of tissue swelling in the lesioned hemispheres (right hemispheres) is presented in Figure 3. The value of brain swelling in the sham group was near to zero. MCA occlusion of the right hemispheres in control ischaemic rats developed tissue swelling after 24 h reperfusion (11.83 ± 2.61%). Pre-treatment with *D. aucheri* extract significantly attenuated the mean value of tissue swelling in the pre-treated ischaemic group (2.67 ± 0.52%) compared to control ischaemic group (*p* = 0.004).

### The pre-treatment effects of *D. aucheri* extract on the MDA and NOx contents

**Figure 4.**
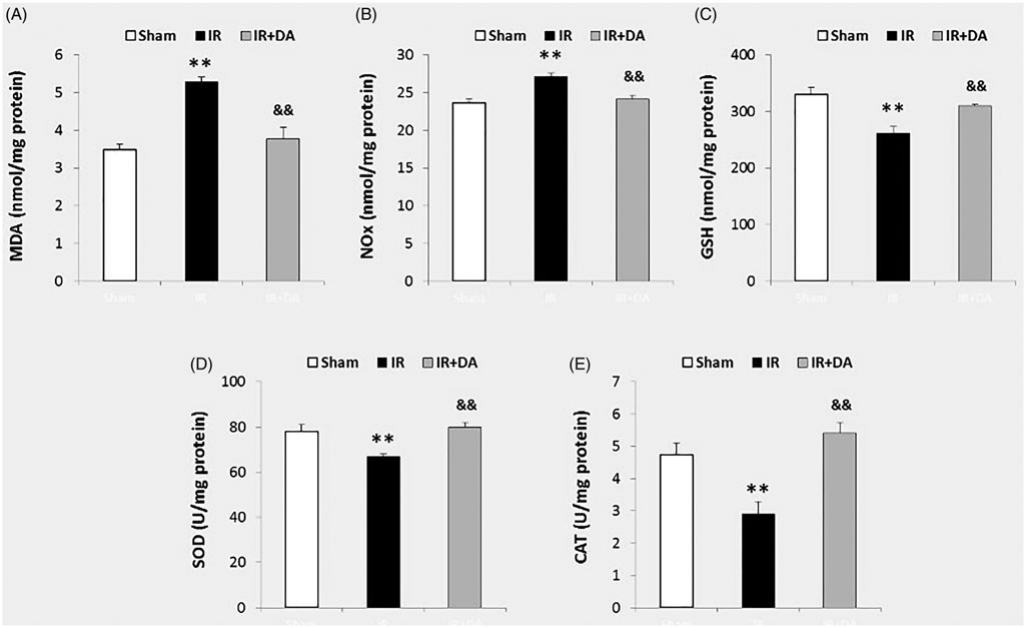
The graphs A, B and C show the contents (nmol/mg protein) of malondialdehyde (MDA), NOx (nitrite and nitrate) and glutathione (GSH), respectively, in the right (ischaemic) hemispheres in sham rats, control ischaemic group (IR) and ischaemic rats treated with *Dorema aucheri* extract (IR + DA) at the end of the experiment. The graphs D and E also show the superoxide dismutase (SOD) and catalase (CAT) activities (U/mg protein), respectively. All values are expressed as mean ± SEM. **As significant difference compared to sham group (*p* < 0.01). ^&&^As significant difference compared to IR group (*p* < 0.01).

MDA and NOx contents of the right hemispheres at the termination of the experiment are presented in Figure 4. The data of ischaemic hemispheres revealed that MCA occlusion significantly increased the MDA levels in control ischaemic group (5.30 ± 0.11 nmol/mg protein) compared with sham group (3.49 ± 0.15 nmol/mg protein), (*p* = 0.0003). Occlusion of MCA also significantly enhanced the levels of NOx in control ischaemic group (27.16 ± 0.49 nmol/mg protein) compared with the sham group (23.17 ± 0.46 nmol/mg protein), (*p* = 0.003). Pre-treatment with *D. aucheri* extract significantly decreased the levels of MDA and NOx in pre-treated ischaemic group (3.78 ± 0.31 nmol/mg protein and 24.11 ± 0.59 nmol/mg protein, respectively) compared to control ischaemic group, (for MDA *p* = 0.001 and NOx *p* = 0.004).

### The pre-treatment effects of *D. aucheri* extract on glutathione content

The glutathione levels (nmol/mg protein) of the right hemispheres are shown in Figure 4 at the termination of the experiment. Occlusion of MCA significantly decreased glutathione levels of the lesioned hemispheres in control ischaemic rats (262 ± 11 nmol/mg protein) compared with the sham group (331 ± 12 nmol/mg protein), (*p* = 0.001). Pre-treatment with *D. aucheri* extract significantly enhanced the levels of glutathione in pre-treated ischaemic group (310 ± 3 nmol/mg protein) compared to control ischaemic group (*p* = 0.009).

### The pre-treatment effects of *D. aucheri* extract on the activities of SOD and catalase

The relative activities of SOD and catalase in the right hemispheres are presented in Figure 4 after 24 h reperfusion. The results of ischaemic hemispheres indicated that MCA occlusion significantly decreased the activity of SOD in control ischaemic group (67 ± 01 U/mg protein) compared to sham group (78 ± 3 U/mg protein), (*p* = 0.001). Occlusion of MCA also decreased the catalase activity of control ischaemic group (2.9 ± 0.3 U/mg protein) compared with sham group (4.7 ± 0.3 U/mg protein), (*p* = 0.013). *Dorema aucheri*-pre-treated ischaemic rats showed a significant increase in the values of SOD and catalase activities (80 ± 2 U/mg protein and 5.4 ± 0.3 U/mg protein, respectively) compared to control ischaemic group, (for SOD *p* = 0.0002 and for catalase *p* = 0.002).

### Morphological examination of neuronal damage

**Figure 5.**
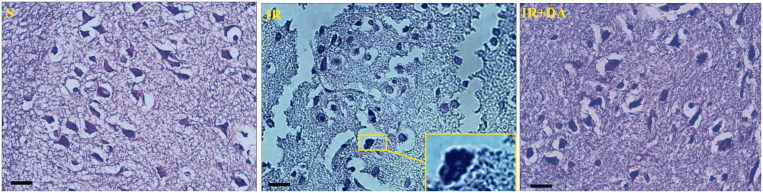
The photomicrographs illustrating the Cresyl violet-stained coronal sections from right hemispheres of sham (S), control ischaemic (IR) and *Dorema aucheri*-treated ischaemic (IR + DA) groups. The enlarged rectangle at a stained section of the control IR group at higher magnification (1000×) indicates apoptotic bodies and pyknotic nuclei with shrunken appearance (Scale bar = 20 µm, 400×).

In order to evaluate the morphologies of the neuronal damage in more detail, the cresyl violet-stained coronal sections were examined at 400× magnification (Figure 5). Normal cells, particularly neurons, in sham rats stained lightly with cresyl violet and had dispersed chromatin with normal nuclei in cortex and striatum of the stained sections. In contrast to sham rats, a large number of apoptotic cells were observed in ischaemic regions of control ischaemic rats that were characterized with apoptotic bodies (darkly stained), pyknotic nuclei and shrunken appearance of nucleus and cytoplasm. The nucleus of apoptotic cells was appeared to have prominent spherical apoptotic bodies and condensed chromatin. Treatment with *D. aucheri* considerably decreased the apoptotic markers in cortical and striatal areas of the ischaemic hemispheres in treated ischaemic groups.

## Discussion

According to the powerful antioxidant effects of *D. aucheri* extract and the crucial role of oxidative damage in pathophysiology of ischaemic stroke (Khanahmadi et al. 2012; Khoshvaghti et al. 2013; Rodrigo et al. 2013), the current study examined the pre-treatment effects of *D. aucheri* extract on cerebral ischaemia-reperfusion injury in an experimental model of MCA occlusion. Our findings revealed the neuroprotective roles of *D. aucheri* extract on brain infarction and swelling. Our results also showed that pre-treatment with this extract could attenuate the oxidative and nitrosative damage in the ischaemic hemispheres of the pre-treated ischaemic rats. Furthermore, *D. aucheri* extract prevented weakening of the brain antioxidant defence system in ischaemic animals. Hence, it is concluded that pre-treatment with *D. aucheri* extract can protect the ischaemic brain against ischaemia-reperfusion injury possibly by potentiation of the brain antioxidant capacity and inhibition of oxidative and nitrosative damage.

The findings of the present study revealed an increase in the levels of ROS and reactive nitrogen species, RNS (an increase in the levels of MDA and NOx) after cerebral ischaemia-reperfusion injury (Figure 4). Overproduction of these poisonous free radicals has a crucial role in the pathogenesis of brain damage after ischaemic stroke (Allen and Bayraktutan 2009; Rodrigo et al. 2013). ROS and RNS generation during cerebral ischaemia or cerebral blood flow restoration in reperfusion phase impairs the structures of cellular macromolecules in the form of protein oxidation and lipid peroxidation, which ultimately leads to cell death (Olmez and Ozyurt 2012; Rodrigo et al. 2013). Hence, scavenging or inhibition of these free radicals by using the different antioxidants would attenuate the injuries of cerebral ischaemia-reperfusion. Since *D. aucheri* extract has the powerful antioxidant properties and it can potentiate the antioxidant capacity of living tissues (Khanahmadi et al. 2012; Khoshvaghti et al. 2013), pre-treatment with this extract in the present study noticeably attenuated the cerebral ischaemia-reperfusion injury including cerebral infarction and swelling (Figures 2 and 3). Based on available documents, *D. aucheri* extract contains many medicinal properties and significant amounts of the antioxidant compounds such as flavonoids, anthocyanins and phenolic acid (Khanahmadi et al. 2012; Mianabadi et al. 2015). These constituents are a category of highly effective natural antioxidant compounds that have the effective antioxidant properties (Moghadas et al. 2012). It has been reported that the main antioxidant function of *D. aucheri* extract may be associated with flavonoids constituents (Mianabadi et al. 2015). Flavonoids can mediate neuroprotective activities in several pathological states due to the antioxidant properties of these constituents in avoiding poisonous free radical-induced oxidative damage (Gutierrez-Merino et al. 2011). So, it is suggested that pre-treatment by *D. aucheri* in ischaemic rats has decreased the oxidative and nitrosative damage (decrement of MDA and NOx contents) due to containing the flavonoids constituents. Moreover, flavonoids can exert other neuroprotective effects in several neurodegenerative diseases, including the antiapoptotic and anti-inflammatory effects (Gutierrez-Merino et al. 2011; Magalingam et al. 2015). The neuroprotective activities of other constituents of *D. aucheri* extract such as anthocyanins and phenolic acid have also been reported in various neurodegenerative diseases (de Pascual-Teresa 2014; Strathearn et al. 2014; Zhou et al. 2017; Gol et al. 2017). Therefore, it is concluded that the neuroprotective nature of *D. aucheri* extract in pre-treated ischaemic rats may be related to the mentioned constituents during cerebral ischaemia-reperfusion injury. In this regard, other beneficial properties of *D. aucheri* extract have been reported by previous studies. The hepatoprotective effects of *D. aucheri* extract have been reported in liver injury induced by carbon tetrachloride (CCl_4_) in rats (Sadeghi et al. 2007). *Dorema aucheri* extract has also shown antihyperglycemic, antihyperlipidemic and hepatoprotective properties in an experimental model of type-2 diabetes in rats (Ahangarpour et al. 2014). It noticeably improved the hepatic enzymes activities as well as insulin and leptin levels in diabetic rats.

The capacity of the brain antioxidant system is weakened following brain ischaemia that exacerbates the ischaemia-reperfusion injury through generation of the toxic free radicals of ROS and development of tissue oxidative damage (Kondo et al. 1997; Slemmer et al. 2008; Chen et al. 2011). In this regard, our results indicated that occlusion of MCA decreased the activities of SOD and catalase in the ischaemic hemispheres. These enzymes are two typical endogenous components of the antioxidant defence system of the brain cell (neurons) against oxidative stress. Hence, a decline in the activities of SOD and catalase has been reported to exacerbate the neuronal cell injury and cerebral infarction following ischaemic stroke (Kondo et al. 1997; Gu et al. 2004). On the other hand, our results revealed that pre-treatment with *D. aucheri* extract prevented the decreasing of the activities of SOD and catalase in the ischaemic hemispheres. In this regard, the antioxidant activity of *D. aucheri* extract has been reported previously (Khanahmadi et al. 2012). Additionally, Khoshvaghti et al. (2013) demonstrated that administration of *D. aucheri* extract for 14 days increased the serum levels of SOD and glutathione peroxidase (GPX) activities in normal rats. SOD is responsible for in the process of ROS elimination by reducing superoxide anion to hydrogen peroxide (Kondo et al. 1997). Catalase is also considered an essential enzyme in the process of ROS elimination by decomposing hydrogen peroxide to H_2_O and O_2_ (Gu et al. 2004). Our results also showed that pre-treatment with *D. aucheri* extract prevented the decreasing of the glutathione content of the ischaemic brains. Cellular glutathione has an essential role in neuronal protection against oxygen free radicals (ROS) in cerebral ischaemia-reperfusion injury (Song et al. 2015). Hence, a decline in the intracellular glutathione levels in the ischaemic brain can induce apoptotic cascades by releasing the cytochrome C from mitochondria (Schafer and Buettner 2001). Therefore, in accompany with potentiation of the endogenous antioxidant defence system of ischaemic hemispheres, the levels of oxidative damage (reduced MDA and NOx contents) decreased in the pre-treated ischaemic rats with *D. aucheri* extract. Hence, it is concluded that pre-treatment with *D. aucheri* extract can attenuate the ischaemia-induced brain injuries by reduction of oxidative damage and potentiation of the endogenous antioxidant defence system.

Beside the cytoprotective effects of *D. aucheri* extract, the harmful effects of this extract must be considered. It has been shown that the hydroalcoholic extract of *D. aucheri* leaves showed hepatotoxicity with concomitant changes of some liver enzymes when it was injected intraperitoneally at different doses in mice (Mostafavi et al. 2013). Nonetheless, hepatoprotective activity of the aqueous extract of *D. aucheri* leaves has been reported against CCl_4_-induced liver damage when it was administered orally in rats (Sadeghi et al. 2007). *Dorema aucheri* extract has also improved the hepatic enzymes activities and hepatocytes function in diabetic rats (Ahangarpour et al. 2014). It appeared that the manner of administration as well as the type of extraction (aqueous or hydroalcoholic) may be important in these controversial results. Hence, more studies must be done to clarify the other protective or harmful activities of *D. aucheri* extract.

## Conclusions

*Dorema aucheri* extract has neuroprotective effects and it can protect against the cerebral ischaemia-reperfusion injury such as infarction and swelling. Pre-treatment with this extract prevents weakening of the brain antioxidant defence system during cerebral ischaemia-reperfusion injury and inhibits the oxidative and nitrosative damage at ischaemic brain. It is suggested that potentiation of the antioxidant capacity of ischaemic brain by *D. aucheri* extract is one of the principal mechanisms of neuroprotection during cerebral ischaemia-reperfusion injury.
